# β-Glucan Induces Training Immunity to Promote Antiviral Activity by Activating TBK1

**DOI:** 10.3390/v15051204

**Published:** 2023-05-19

**Authors:** Guolei Wang, Zhiqiang Li, Mingfu Tian, Xianghua Cui, Jun’e Ma, Siyu Liu, Chenglin Ye, Li Yuan, Muhammad Suhaib Qudus, Uzair Afaq, Kailang Wu, Xinghui Liu, Chengliang Zhu

**Affiliations:** 1 Department of Clinical Laboratory, Institute of Translational Medicine, Renmin Hospital of Wuhan University, Wuhan 430060, China; 2 State Key Laboratory of Virology, College of Life Sciences, Wuhan University, Wuhan 430072, China; 3 Department of Clinical Laboratory, Zhongnan Hospital of Wuhan University, Wuhan 430060, China; 4 Department of Clinical Laboratory, Shanghai Gongli Hospital, The Second Military Medical University, Pudong New Area, Shanghai 200135, China

**Keywords:** trained immunity, innate immunity, TBK1, ubiquitination

## Abstract

Many studies have shown that β-glucan induces a trained immune phenotype in innate immune cells to defend against bacterial and fungal infections. The specific mechanism involves cellular metabolism and epigenetic reprogramming. However, it is unclear whether β-glucan plays a role in antiviral infection. Therefore, this study investigated the role of trained immunity induced by *Candida albicans* and β-glucan in antiviral innate immunity. It showed that *C. albicans* and β-glucan promoted the expression of interferon-β (IFN-β) and interleukin-6 (IL-6) in mouse macrophages triggered by viral infection. In addition, β-glucan pretreatment attenuated the pathological damage induced by the virus in mouse lungs and promoted the expression of IFN-β. Mechanistically, β-glucan could promote the phosphorylation and ubiquitination of TANK Binding Kinase 1 (TBK1), a key protein of the innate immune pathway. These results suggest that β-glucan can promote innate antiviral immunity, and this bioactive material may be a potential therapeutic target for antiviral treatment.

## 1. Introduction

Innate immunity plays a crucial role in the fight against pathogenic infections. Previously, it was thought that immune memory exclusively exists in adaptive immunity. However, a number of studies are increasingly showing that innate immunity also has memory. Innate immune cells develop a certain memory after the first exposure to an antigen and evoke a stronger immune response the next time they are exposed to the same antigen or a heterologous antigen, which is called trained immunity (TI) [1].TI can be induced by different stimuli, including Bacillus Calmette-Guérin (BCG) [2], *C. albicans* (Cal) [3], β-glucan [4,5], and lipopolysaccharide (LPS) [6]. All of them can induce enhanced anti-bacterial immune responses in innate immune cells and stem cells. It has been found that the influenza A virus can also induce trained immunity of lung-resident macrophages against infection by *Streptococcus pneumoniae* [7]. However, whether TI modulates antiviral immunity needs to be further investigated.

Pattern recognition receptors (PRRs) play a crucial role in the recognition of viral infections and in the activation of subsequent innate immune responses [8]. Different PRRs such as Toll-like receptor 3 (TLR3), a retinoic acid-inducible gene I (RIG-I), melanoma differentiation-associated protein 5 (MDA5), and cyclic GMP-AMP synthase (cGAS) and others sense viral invasion and activate respective Toll/Interleukin-1 receptor (TIR) domain-containing adapter-inducing interferon-β (TRIF), mitochondrial antiviral-signaling protein (MAVS), and stimulator of interferon genes (STING), which subsequently transduces the activation signal to the downstream TANK Binding Kinase 1 (TBK1), triggering a signaling cascade that induces the production of type I interferons and pro-inflammatory cytokines. Thus, TBK1 is an important hub in the antiviral innate immune signaling pathway and is critical for the production of type I interferon. Ubiquitination and deubiquitination are important modifications of TBK1 activation. E3 ubiquitin ligase RNF128 promotes DNA- and RNA virus-induced K63-linked polyubiquitination of TBK1 [9]. Scavenger receptor A, expressed mainly in macrophages, can deubiquitinate TBK1 by promoting the aggregation of USP15, a member of the cysteine protease deubiquitylases, thereby inhibiting DNA and RNA virus-induced IFN production [10].

Previous studies have reported that trained immunity primarily mediates epigenetic modifications or metabolic reprogramming of innate immune cells to modulate inflammatory responses, but whether it has a direct effect on the antiviral innate immune pathway needs to be further investigated. Here, we found that the “training” of macrophages by *C. albicans* and β-glucan is also involved in the regulation of immune responses triggered by RNA and DNA viruses. Consistently, β-glucan-treated mice were more resistant to the virus and displayed a more intense inflammatory response. Mechanistically, *C. albicans*/β-glucan-mediated trained immunity promotes antiviral native immunity by modulating TBK1 ubiquitination. Together, this study reveals a novel mechanism by which trained immunity regulates antiviral immunity through post-translational modifications, which may provide new therapeutic targets for antivirals.

## 2. Materials and Methods

### 2.1. Cells

The peritoneal macrophage (PM) was obtained from the peritoneal cavity of C57BL/6 mice according to a standard protocol [11]. Briefly, 1 mL of 3% mercaptoacetate broth was injected intraperitoneally into the peritoneal cavity of each mouse. Normal feeding was performed for 4 days. Mice were euthanized by rapid cervical dislocation. Mice were soaked in 70% alcohol, and then the skin was removed along the midline of the abdomen with sterile scissors to expose the intact peritoneum. Using a syringe, 7–10 mL of PBS was injected into the peritoneal cavity of the mice, followed by gentle shaking of the abdomen to aspirate the fluid from the peritoneal cavity. After this, the fluid was transferred to a 15 mL centrifuge tube and centrifuged at 1500 rpm/min for 5 min. Then, cells were seeded into 12-well plates (2–5 × 10^5^) or 6-well plates (1 × 10^6^) with Dulbecco’s modified Eagle medium (DMEM) (Gibco, Grand Island, NY, USA) containing 10% Fetal Bovine Serum (FBS) and 100 U/mL penicillin, and 100 μg/mL streptomycin sulfate. Cells were cultured in a humidified incubator at 37 °C with 5% CO_2_.

### 2.2. Treatment of PMs with Different Stimuli

β-glucan was purchased from Sigma-Aldrich (Sigma-Aldrich, St. Louis, MO, USA). Vesicular stomatitis virus (VSV) and herpes simplex virus type 1 (HSV-1) were presented by Bo Zhong from Wuhan University. LPS, poly(I:C), and poly(dA:dT) were purchased from invivoGen (InvivoGen, San Diego, CA, USA). Cells were pretreated with β-glucan (10 μg/mL), heat-inactivated Cal (1 × 10^4^ CFU/mL), LPS (100 ng/mL), and poly(I:C) (100 ng/mL) for 24 h. The supernatant was discarded, and cells were washed twice with PBS, followed by a period of 6-day culture with DMEM containing 10% FBS. Cells were subsequently stimulated with LPS (1 μg/mL) for 4 h, poly(I:C) (1 μg/mL), and poly(dA:dT) (2 μg/mL) for 8 h, VSV (1 × 10^4^ PFU/mL) and HSV-1 (2 × 10^4^ PFU/mL) for 8 h before the collection of samples.

### 2.3. Mice

C57BL/6 WT mice were purchased from the Hubei Research Center of Laboratory Animals (Wuhan, China). All animal experiments were approved by the IACUC of State Key Laboratory of Virology, College of Life Sciences, Wuhan University, and all animal studies were conducted in accordance with the Animal Welfare Act and the National Institutes of Health guidelines for the care and use of experimental animals in biomedical research. Male mice who were 10 weeks old were injected i.p. with β-glucan (1 mg/each) or an equal volume of pyrogen-free phosphate-buffered saline (PBS). Three days later, re-injection was performed. On the 7th day, mice were injected i.p. with HSV-1 viruses (1 × 10^7^ PFU/each).

### 2.4. RNA Extraction, cDNA Synthesis, and Quantitative RT-PCR

From tissues or cells, total RNA was extracted with Trizol reagent (Invitrogen, San Diego, CA, USA), and cDNA was generated from 1 µg of RNA with reverse transcription mix (Vazyme Biotech Co., Ltd., Nanjing, China). The products of reverse transcription were used as the template for amplification, and real-time PCR was conducted using ChamQ SYBR qPCR Master Mix (Vazyme Biotech Co., Ltd., China) and specific primers based on the experimental needs. All primers used in this study are listed in Table 1.

### 2.5. ELISA

The concentrations of IFN-β in sera were measured by ELISA Kits (Invitrogen, San Diego, CA, USA).

### 2.6. Western Blotting

Electrophoresis was performed with 6–10% SDS-PAGE gels. After that, the proteins were transferred to PVDF membranes and blocked with 5% non-fat milk for 45 min. The membranes were washed 3 times with Tris Buffered Saline with Tween (TBST), followed by incubation overnight with primary antibodies at 4 °C. After 3 times washing with TBST, the membranes were incubated with secondary antibodies (Jackson ImmunoResearch, Pennsylvania, PA, USA) (1:5000 dilutions) for 45 min and then washed 3 times with TBST. Protein bands were detected by exposure.

For immunoprecipitation, total protein was extracted using NP40 lysate containing protease inhibitor (50 mm Tris-HCl (pH 7.5), 0.5 mm EDTA, 150 mM NaCl, 1% NP40, and 1% SDS). Cell lysates were co-incubated with specific antibodies and protein A/G agaroses overnight at 4 °C. The target proteins were detected using SDS-PAGE gel electrophoresis with specific antibodies.

The primary antibodies used in the study were as follows: mouse anti-p-TBK1 (Abcam, Cambridge, UK, 1:2000), mouse anti-TBK1 (Abcam Cambridge, UK and Abclonal, Boston, MA, USA, 1:2500), anti-GAPDH (Sigma-Aldrich, MO, USA, 1:5000), anti-K63 (Cell Signaling Technology, Danvers, MA, USA, 1:2000), anti-K48 (Cell Signaling Technology, Danvers, MA, USA, 1:2000), and anti-Ubiquitin (Cell Signaling Technology, Danvers, MA, USA, 1:2000).

### 2.7. Histopathology

We fixed mice lung samples in 4% paraformaldehyde and used an automatic paraffin embedding machine to make pathological sections after dehydrating, transparency, wax immersion, and embedding. Following H&E staining, histopathological changes were observed under a microscope.

### 2.8. Statistical Analysis

All data are expressed as mean ± SD or mean ± s.e.m. We used two-way analysis of variance (ANOVA) with Holm–Sidak’s multiple comparisons test for multiple comparison groups or unpaired t-tests as appropriate for statistical analysis, and *p* < 0.05 was regarded as a significant result. Statistical analysis was performed using GraphPad PRISM v9.0.0 (GraphPad, San Diego, CA, USA).

## 3. Results

### 3.1. Trained Immunity Induced by β-Glucan Promotes Antiviral Immunity of Macrophages

**Figure 1:**
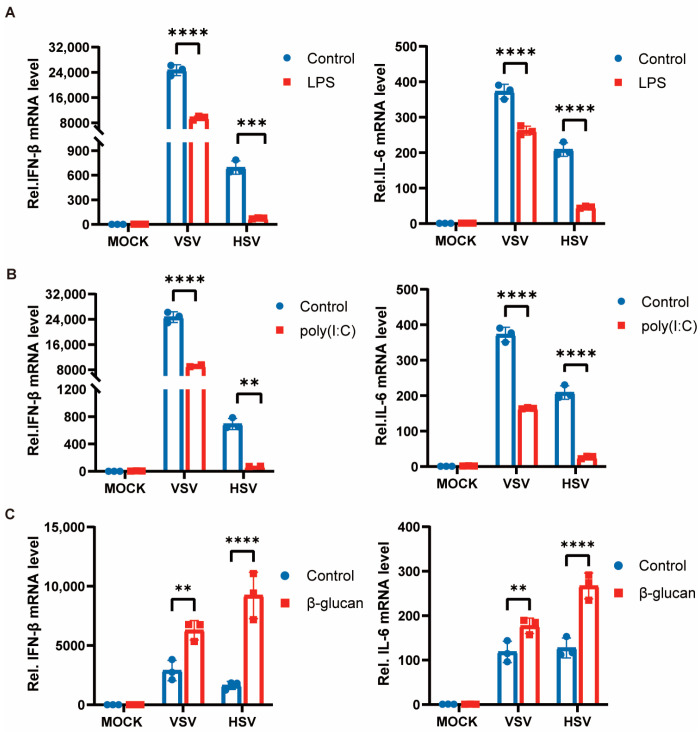
Trained immunity induced by β-glucan promotes antiviral immunity of macrophages. (**A**–**C**) PMs were treated with LPS (**A**), poly(I:C) (**B**), and β-glucan (**C**) for 24 h, washed, and followed by a 6 days rest period, and subsequent infection with VSV and HSV-1 for 8 h. The mRNA levels of IFN-β and IL-6 were detected by RT-PCR. All data were shown as mean ± SD. Experiments were performed with three biological replicates. The asterisk (*) indicates significant differences between the groups. ** *p* < 0.01, *** *p* < 0.001, **** *p* < 0.0001 (Two-way ANOVA followed Holm–Sidak’s multiple comparisons test).

Previous studies have shown that different types of pathogenic microorganisms and their components can induce trained immunity in cells. We specifically selected LPS (gram-negative bacteria cell wall component), poly(I:C) (double-stranded RNA virus analog), and β-glucan (fungi cell wall component) to pretreat peritoneal macrophages for 24 h, and then the stimulation was removed, and the cells were left still for 6 days before secondary stimulation with VSV and HSV-1. The expression of pro-inflammatory cytokines was suppressed after restimulation with the viruses in cells pretreated with LPS and poly(I:C) (Figure 1A,B). However, the training of cells by β-glucan induced an enhancement of cytokine production when restimulated with viruses after 7 days (Figure 1C). These results suggest that the state of immune memory was different after cells were exposed to different pathogenic microbial components. Bacterial and viral pathogens induce a tolerant state, whereas fungi induce a trained immunity against viral infection in macrophages.

### 3.2. Trained Immunity Induced by Candida albicans Facilitates Antiviral Immunity of Macrophages

**Figure 2:**
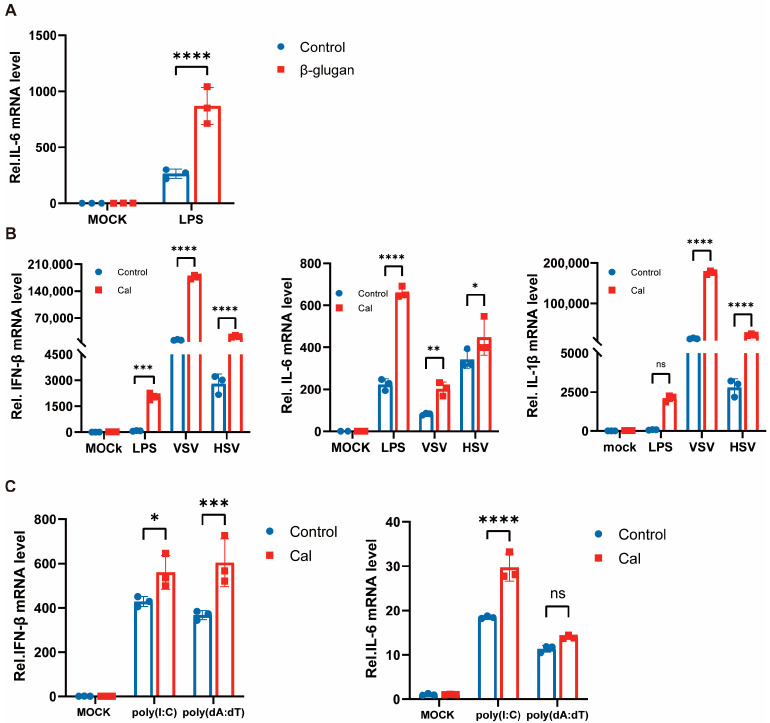
Trained immunity induced by *Candida albicans* facilitates antiviral immunity of macrophages. (**A**) PMs were treated with β-glucan for 24 h, washed, and followed by a 6 days rest period, and subsequent infection with LPS for 4 h. The mRNA levels of IL-6 were detected by RT-PCR. (**B**) PMs were treated with Cal for 24 h, washed, and followed by a 6 days rest period, and subsequent infection with LPS for 4 h, VSV and HSV-1 for 8 h. The mRNA levels of IL-6, IFN-β, and IL-1β were detected by RT-PCR. (**C**) PMs were treated with Cal for 24 h, washed, and followed by a 6 days rest period, and subsequent infection with poly(I:C) and poly(dA:dT) for 8 h. The mRNA levels of IFN-β and IL-6 were detected by RT-PCR. All data were shown as mean ± SD. Experiments were performed with three biological replicates. The asterisk (*) indicates significant differences between the groups. * *p* < 0.05, ** *p* < 0.01, *** *p* < 0.001, **** *p* < 0.0001 (Two-way ANOVA followed Holm-Sidak’s multiple comparisons test).

Previous studies reported that β-glucan promotes cytokine production in the presence of LPS reinfection, and we repeatedly validated this finding in mouse peritoneal macrophages (Figure 2A). As mentioned previously, we also observed enhanced inflammatory factor production in virally reinfected PM cells trained with β-glucan. β-glucan is a major component of the cell wall of *C. albicans* [12], and to determine that the same phenomenon could be observed in macrophages directly exposed to *C. albicans*, we treated cells with heat-inactivated *C. albicans* for 24 h and with LPS, VSV and HSV-1 re-stimulation after 6 days. Similarly, the expression of inflammatory factors IL-6, IL-1β, and IFN-β in macrophages was significantly enhanced (Figure 2B). That is, *C. albicans*/β-glucan-induced trained immunity also promotes type I interferon production and modulates antiviral innate immune responses. Therefore, RNA virus and DNA virus reinfection was mimicked with the viral nucleic acid analogs poly(I:C) and poly(dA:dT), respectively, and the same results were obtained (Figure 2C). These results suggest an important role of trained immunity of PM cells against viral infection and facilitation of virus-mediated production of type I interferon and inflammatory factors, which is a broad-spectrum phenomenon, i.e., the same effect for viruses of different nucleic acid types.

### 3.3. Trained Immunity Induced by β-Glucan Regulated TBK1 Activation

**Figure 3:**
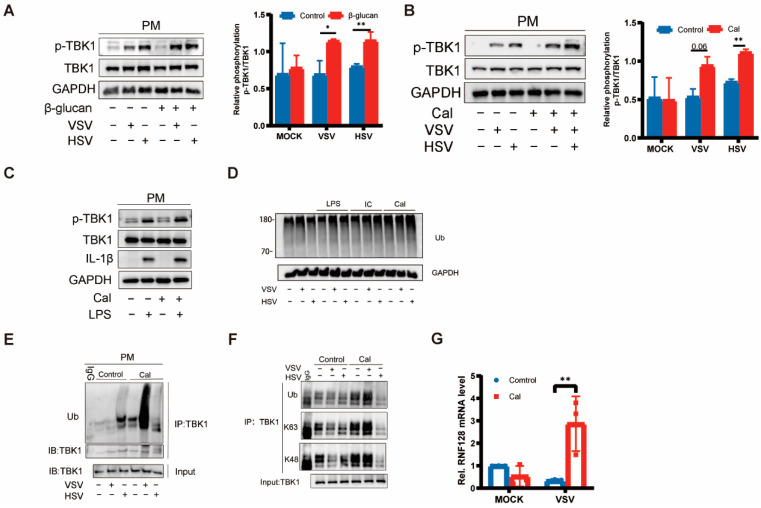
Trained immunity induced by β-glucan regulated TBK1 activation (**A**–**C**) PMs were treated with β-glucan (**A**) and Cal (**B**,**C**) for 24 h, washed, and followed by a 6 days rest period, and subsequent infection with VSV and HSV-1 for 8 h, LPS for 4 h. Western blot of p-TBK1, TBK1 and GAPDH. Band quantification of phosphorylated (p-)TBK1 relative to total TBK1. Data were shown as mean ± s.e.m. (**D**) PMs were treated with LPS, poly(I:C), and Cal for 24 h, washed, and followed by a 6 days rest period, and subsequent infection with VSV and HSV-1 for 8 h. Western blot of ubiquitin. (**E**,**F**) PMs were treated with Cal for 24 h, washed, and followed by a 6 days rest period, and subsequent infection with VSV and HSV-1 for 8 h. Co-immunoprecipitation and immunoblot analysis of ubiquitin, K63, K48, and TBK1. (**G**) PMs were treated with Cal for 24 h, washed, and followed by a 6 days rest period, and subsequent infection with HSV-1 for 8 h. The mRNA levels of RNF128 were detected by RT-PCR. The asterisk (*) indicates significant differences between the groups. * *p* < 0.05, ** *p* < 0.01 (two-tailed Student’s *t*-test and Two-way ANOVA followed Holm–Sidak’s multiple comparisons test). Data are representative of three independent experiments with similar results (**A**–**F**).

TBK1 is capable of receiving activation signals from multiple PRRs and can be activated by signaling pathways such as TLR3/4-TRIF, RIG-1-MAVS, and cGAS-STING to further phosphorylate IRF3/IRF7 and nuclear factor-kappa B (NF-κB) to initiate the type I interferon production and trigger antiviral native immune responses [13,14,15,16]. Previous studies have shown that the underlying mechanisms of trained immunity involve epigenetic reprogramming and cellular immune metabolism [3,4,5] but do not have a direct impact on the relevant antiviral innate immune pathways. Based on our previous findings, once cells acquired trained immunity, IFN-β production could be promoted in the case of re-stimulation by either DNA-virus, RNA-virus, or LPS. It implies that the regulation of type I interferon production by trained immunity is not pathway-dependent. Given that TBK1 is a key molecule in the natural immune pathway, it is possible that cellular-trained immunity will affect TBK1 activation. Interestingly, we found that both *C. albicans* and β-glucan promoted TBK1 phosphorylation but did not affect the protein levels of TBK1. In other words, TBK1 phosphorylation induced by the virus, representative of enzyme activity, showed a significant trend toward an increase under the influence of β-glucan (Figure 3A,B), suggesting that the trained immunity induced by β-glucan is likely to be related to the post-translational modification of TBK1. However, Despite the enhanced expression of the inflammatory factor IL-1β induced by LPS re-stimulation, the phosphorylation of TBK1 did not change significantly, which suggests that the activation of TBK1 induced by LPS is not dependent on β-glucan pretreatment (Figure 3C). The enhanced expression of inflammatory cytokines induced by LPS may involve epigenomic reprogramming of macrophages [17].

Ubiquitination is crucial for the regulation of TBK1 activity, where ubiquitination of K63 linkage would enhance its activity [18]. Next, we measured TBK1 ubiquitination in PMs after different PAMP pretreatments following viral infection and found that the overall ubiquitination level was not affected (Figure 3C). However, as for TBK1 ubiquitination, *C. albicans* significantly promoted viral infection-induced TBK1 ubiquitination (Figure 3D). Furthermore, we found that β-glucan greatly increased the K63- and K48-linked polyubiquitination of TBK1 induced by the viruses, especially VSV (Figure 3E). As previously described, β-glucan can promote the expression of pro-inflammatory cytokines such as IFN-β, and we hypothesized that this phenomenon is associated with enhanced ubiquitination of TBK1. RNF128 has been reported to interact directly with TBK1 and catalyze K63-linked TBK1 ubiquitination [9]. Here, we examined the mRNA levels of RNF128 and found that induction of cellular trained immunity could promote RNF128 mRNA expression after viral infection (Figure 3F).

### 3.4. β-Glucan Enhances the Antiviral Innate Immunity in Mice

**Figure 4:**
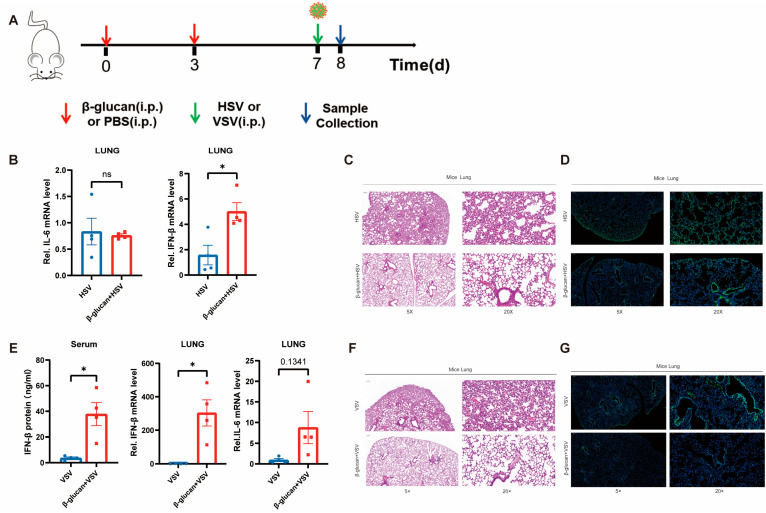
β-glucan enhances the innate antiviral immunity in mice (**A**) WT mice were injected i.p. with β-glucan (1 mg/kg) or PBS 7 d and 4 days prior to injected i.p. with 1 × 10^7^ PFU VSV or HSV-1. Then, collect the Lung tissue after 24 h. (**B**,**E**) The mRNA levels of IFN-β and IL-6 in the mice lung were detected by RT-PCR. The protein levels of IFN-β in mice’s serum were measured by ELISA (**B**). *n* = 4 mice per group. (**C**,**F**) Hematoxylin and eosin staining was performed on the mice’s lung tissue to observe the damaged areas. Scale bars, 200 μm and 50 μm (**D**,**G**) Lung tissues are labeled green by immunofluorescence staining with anti-GFP antibody to observe HSV-1-GFP infection. Immunofluorescence DAPI was used as a nuclear stain (blue). Scale bars, 200 μm and 50 μm. All data were shown as mean ± s.e.m. The asterisk (*) indicates significant differences between the groups. * *p* < 0.05 (Two-way ANOVA (Two-way ANOVA followed Holm–Sidak’s multiple comparisons test).

To confirm the effect of β-glucan-induced trained immunity on viral infection in mice, we treated mice with 1 mg of β-glucan or PBS two times, respectively, and infected them with HSV-1-GFP or VSV-GFP six days later, and collected the lung tissue after 24 h (Figure 4A). It showed that β-glucan pretreatment induced high expression of IFN-β in the mouse lungs but no change in IL-6 expression (Figure 4B,E). Moreover, histopathological results demonstrated that β-glucan pretreatment protected mice from viral infection in the lung. The alveolar structure was more intact with less infiltration of inflammatory cells in mice pretreated with β-glucan (Figure 4C,F). β-glucan–treated mice showed significantly lower HSV-1 infection in the lung compared with PBS mice (Figure 4D,G). In conclusion, β-glucan treatment inhibited the inflammatory response induced by viruses and protected the mice from lung injury caused by viral infection.

## 4. Discussion

β-glucan is a recognized and effective inducer for trained immunity to protect the organism from viral, bacterial, and fungal pathogens [3,19,20,21], and its immunomodulatory effect lasts for several weeks. Training phenotype of macrophages derived from myeloid cells protected the host from the secondary infection and improved the recovery from chemotherapy-induced myeloablation [22]. Exposure of monocytes or macrophages to β-glucan resulted in an enhanced response to stimuli from the same or different pathogens, which is accompanied by changes in chromatin markers such as H3K27ac, H3K4me1 and H3K4me3 [23]. Furthermore, studies show that β-glucan–mediated training immunity state even reverses the immune tolerance state induced by LPS [17]. In this study, we showed that *C. albicans*/β-glucan training significantly enhanced the level of pro-inflammatory cytokines, such as IFN-β, IL-6, and IL-1β, in mouse PMs induced by DNA/RNA virus. In a word, *C. albicans*/β-glucan induced macrophages to produce a trained immune phenotype, thereby may promote an antiviral natural immune response.

Host recognition of invading pathogens by PRR induces the initiation of innate immunity to produce large amounts of IFN [8]. The recognition of β-glucan is mainly through the C-type lectin receptordectin 1 [24].TBK1 plays an important role in antiviral natural immunity as a phosphokinase necessary for the type I interferon pathway [25]. In turn, post-translational modifications of TBK1, especially phosphorylation and ubiquitination, are essential for the regulation of its activity. Phosphorylated TBK1 can amplify the cascade by activating more TBK1 through trans-autoreactivation [26]. In addition, TBK1 ubiquitination also affects TBK1 activation. TRAF3IP3 mediates TBK1 degradation by promoting K48 ubiquitination of TBK1, thereby inhibiting RNA virus-induced IFN production [27]. In contrast, RNF128-mediated K63 ubiquitination of TBK1 promotes the activation of TBK1 [9]. Our results suggest that the training effect of *C. albicans*/β-glucan promotes TBK1 phosphorylation and is achieved by promoting TBK1 ubiquitination.

It has been demonstrated that β-glucan can improve the defense of experimental mice against pathogenic infections [3,28]. Glucan feeding significantly reduced the effects of influenza infection on total mortality by activating both cellular and humoral immune responses, resulting in a lower level of viral load [29]. β-glucan have the potential to benefit the development of drugs against novel coronaviruses by reducing inflammatory responses and antioxidation in cells [30]. Moreover, β-glucan-trained has shown promising therapeutic effects in clinical respiratory diseases as an adjuvant for vaccines [31]. In this study, β-glucan significantly enhanced the expression of IFN in the lungs of mice. Furthermore, our results indicated that β-glucan reduced viral infection and reduced lung tissue damage in mice. The results of the present analysis are not free of limitations. Most of the conclusions are based on measuring mRNA rather than protein levels. It is necessary to validate the results using another experiment in the future.

In conclusion, this study explains a unique mechanism in trained immune regulation of cellular antiviral responses induced by DNA and RNA virus infections, providing new targets for the development of future antiviral drugs or vaccines.

## Figures and Tables

**Table 1:** Primer sequence of qPCR.

Gene	Forward (5′ → 3′)	Reverse (5′ → 3′)
m-IL-6	TTCCATCCAGTTGCCTTCTTG	AATTAAGCCTCCGACTTGTGAA
m-IFN-β	AGATCAACCTCACCTACAGG	TCAGAAACACTGTCTGCTGG
m-RNF128	AGCTTCCATAATAAACACCT	CCTTTAACTGCCTCTGTAATAA
m-GAPDH	GAA GGG CTC ATG ACC ACA GT	GGA TGCAGG GAT GAT GTT CT
m-IL-1β	GTTCCCATTAGACAACTGCACTACAG	GTCGTTGCTTGGTTCTCCTTGTA

## Data Availability

Not applicable.
